# “I Was a Full Time Proper Smoker”: A Qualitative Exploration of Smoking in the Home after Childbirth among Women Who Relapse Postpartum

**DOI:** 10.1371/journal.pone.0157525

**Published:** 2016-06-16

**Authors:** Sophie Orton, Tim Coleman, Sarah Lewis, Sue Cooper, Laura L. Jones

**Affiliations:** 1 UK Centre for Tobacco & Alcohol Studies & Division of Primary Care, University of Nottingham, Nottingham, United Kingdom; 2 UK Centre for Tobacco & Alcohol Studies & Division of Epidemiology & Public Health, University of Nottingham, Nottingham, United Kingdom; 3 UK Centre for Tobacco & Alcohol Studies & Institute for Applied Health Research, University of Birmingham, Birmingham, United Kingdom; University of Manchester, UNITED KINGDOM

## Abstract

**Background:**

Many women stop smoking during pregnancy but relapse shortly afterwards, potentially putting their infants at risk of secondhand smoke (SHS) exposure. Women who were able to stop during pregnancy may be a motivated group, receptive to making behaviour changes postpartum to protect their infant from SHS exposure. Understanding more about their experiences of relapse, and if this influences home smoking behaviours and children’s exposure to SHS in the home may help to inform intervention development to prevent infant SHS exposure.

**Methods:**

Guided by interpretative phenomenological methodology we conducted and analysed nine semi-structured interviews with women who quit smoking during pregnancy, but relapsed ≤3 months postpartum.

**Findings:**

Central to mothers’ accounts of their smoking behaviours during pregnancy and postpartum was their desire to be a ‘responsible mother’. Mothers described using strategies to protect their infant from SHS exposure, and held strong negative attitudes towards other smoking parents. After relapsing, mothers appeared to reposition themselves as ‘social’ or *‘occasional’ smokers rather than ‘regular’ smokers*.

**Conclusions:**

Findings suggest that interventions to prevent/reduce infants' home SHS exposure should build on mothers' intentions to be responsible parents. As mothers who relapse principally view themselves as ‘social’ or ‘occasional’ smokers, interventions that are highlighted as relevant for women with these types of smoking patterns may be more likely to be responded to, and, ultimately, be effective.

## Background

Over half (54%) of women manage to quit smoking before or during pregnancy; however, a reported 70% of these women relapse in the first six months postpartum.[1,2] Relapse rates are particularly high in the initial postpartum period, with just under 50% of pregnancy quitters relapsing within the first 6 weeks after giving birth.[3] This accounts for 66% of all pregnancy quitters who will relapse. Maternal smoking is one of the primary sources of child secondhand smoke (SHS) exposure in the home, [4] and consequently postpartum relapse has potentially important implications for infant and child SHS exposure.

Postpartum smoking relapse has been found to be associated with increased deprivation, [3,5] being single, [5] higher parity, [3,5] not breastfeeding, [3,5] stress, [6] and intending to quit only for pregnancy. [6] Qualitatively, women report that the stress of caring for a new baby, no longer needing to protect the baby from their smoking, adjusting to their new identity as a mother and social influences of smoking (e.g. smoking friends or partners) are important factors in postpartum smoking relapse. [7] The presence of other smokers in the household, [3,5,6] and in particular living in a home where smoking is permitted indoors [6,8] are also important risk factors for postpartum smoking relapse. Women who relapse are therefore less likely to have a smoke-free home (SFH), putting their infants at a further increased risk of SHS exposure.

Although smoking in the home and relapse are linked, little is currently known about why women who have managed to stop smoking during pregnancy may start again, and what their home smoking behaviours are following relapse. Understanding more about this is important, as women who manage to quit smoking for at least part of their pregnancy are a potentially motivated group who may be receptive to making behaviour changes to protect their baby from potential SHS exposure. [9–11] Women who continue to smoke during pregnancy may be less receptive to making changes to protect their child from SHS exposure; research has found these women are less likely to engage in other positive antenatal health behaviours, [12,13] less likely to feel personally responsible for the health of their baby in-utero, [12] and contest public health discourses of the risks associated with smoking. [14,15] Conversely, women who abstained from smoking during pregnancy were motivated and able to engage in positive behaviour changes to protect their baby from smoke exposure in utero, with concern for baby’s health and not wanting to be a smoking role model for their children being key motivations for stopping during pregnancy.[9] Self-efficacy, which has been identified as an important construct predicting smoking cessation and maintenance of behaviour change, [16,17] may also be high among this group of women having successfully quit smoking during their pregnancy.

Pregnancy and parenthood have been identified as key ‘teachable moments’, defined as naturally occurring life transitions in which individuals are more likely to be successful in positive health behaviour changes.[18,19] The early postpartum period, around the time when women are at risk of relapse, may therefore be an ideal time to intervene to reduce or prevent SHS exposure in the home by harnessing these mothers’ intrinsic motivation and self-efficacy to maintain their quit attempt. This study explored why women who stopped smoking in pregnancy re-started again afterwards, with a particular focus on how this affected their home smoking behaviours and whether relapsing led to infant SHS exposure in the home.

## Methods

### Qualitative methodology

This research was conducted using the principles of interpretative phenomenological analysis (IPA), [20] an inductive approach that aims to explore how participants interpret and make sense of their world, and formulate their own biographical stories. [20–23] This approach was considered appropriate as it was important to understand smoking behaviour from women’s perspectives; exposure in infants and children is a sensitive, complex and changeable issue, and mother’s experiences of smoking are likely to be unique to the individual. This study is reported using the COREQ guidelines (S1 Table).[24]

### Ethical approval

The study received a favourable opinion by Derbyshire Research Ethics Proportionate Review Sub-Committee (reference number 11/EM/0078).

### Recruitment

**Fig 1:**
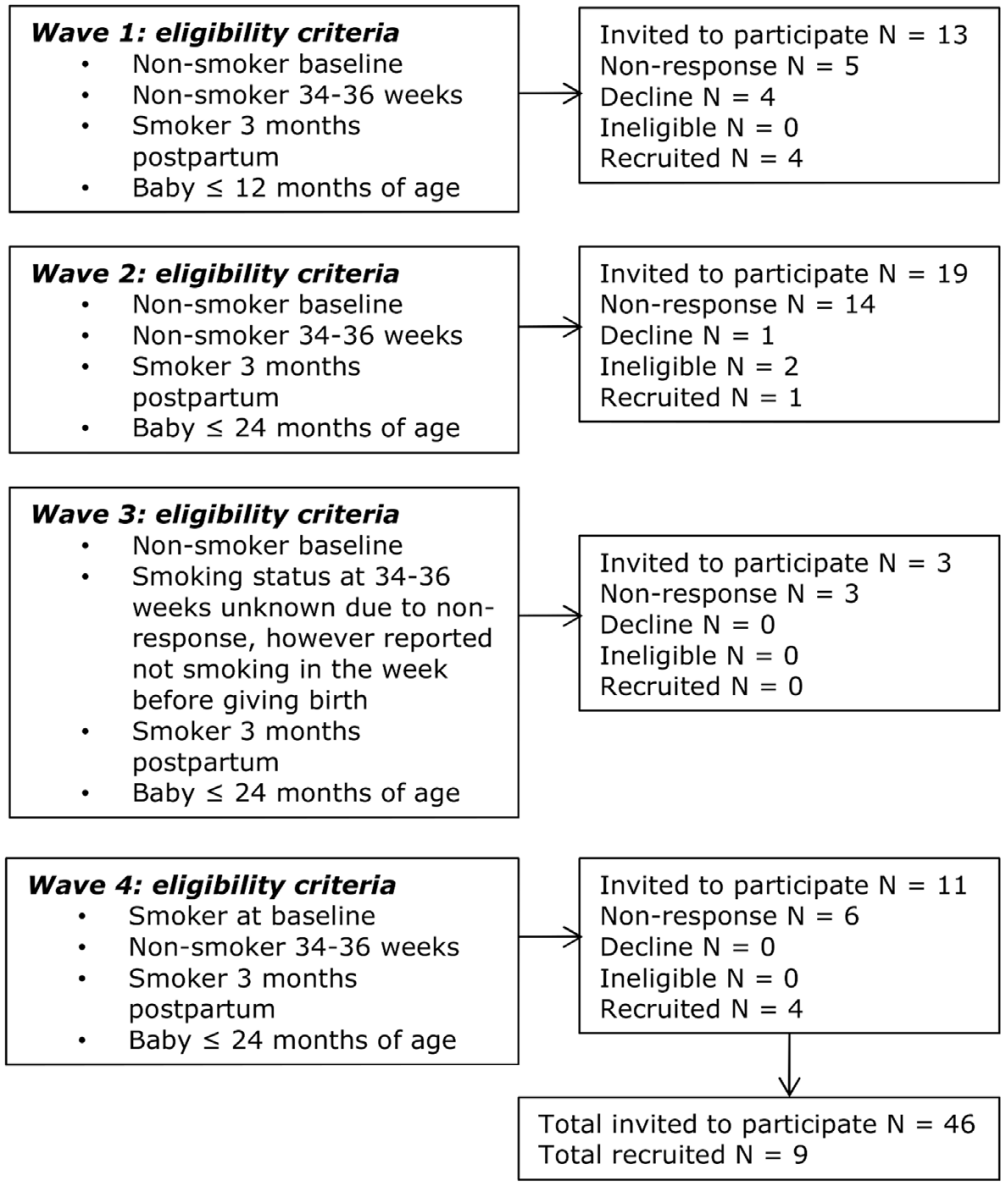
Recruitment waves and response rates.

Women were recruited from the Pregnancy Lifestyle (PLS) cohort.[25] The PLS is a longitudinal pregnancy cohort recruited within Nottingham, England, which collected detailed information on smoking behaviour across pregnancy and the early postpartum period. [25] In line with IPA methodology [20] a sample size of up to 10 participants was identified as appropriate. Four recruitment waves of women who had reported that they had stopping smoking for at least some of their pregnancy but were smoking again at 3 months post-delivery were conducted (Fig 1). Women were excluded from participation if they reported themselves to be currently pregnant.

Women were invited to participate by post, and contacted by telephone or text message thereafter. Invitation letters provided women with details of how they could get in contact if they were interested in participating, and enclosed a copy of the participant information sheet. In total, 46 participants were invited to interview across four waves, with 9 participants consenting to take part (Fig 1).

### Data collection methods

All interviews were conducted by SO, who is female and has a background in health psychology and smoking in pregnancy. Written informed consent was obtained prior to interview. Interviews were carried out in participants’ homes, and lasted on average 40 minutes (range 10–60 minutes). All interviews were audio recorded and transcribed verbatim. Participants were offered an inconvenience allowance of a £20 shopping voucher as compensation for their time. Basic demographic details were collected via questionnaire.

### Interview schedule

The interview schedule was developed using literature, discussion and reviewed by and piloted with a member of the public who was a current smoker and had smoked during two previous pregnancies. The interview schedule covered mother’s experiences of smoking during pregnancy, smoking relapse, smoking in their home and their attitudes towards child and infant SHS exposure. The schedule was designed to be semi-structured to allow flexibility and further exploration of areas of interest. [22]

## Analysis

In line with the guidance for IPA research, [22] a three-step approach was taken to analysis. Firstly, each transcript was read and re-read; comments, associations, observations, interpretations and preliminary themes were noted. Secondly, connections between these preliminary themes were examined; these were then clustered and a coherent list of subordinate and superordinate themes was drawn. Finally, this process was repeated across all transcripts. A consolidated master list of themes was constructed based upon prevalence within the data and the richness of the examples. Themes were continually checked against the data to ensure accurate interpretation of the text, as were new themes that emerged from later transcripts.

The analysis was conducted by SO and LLJ. Themes were discussed within the wider authorship to ensure clarity, plausibility and appropriate interpretation of the data. Contradictory accounts were explored and reported where appropriate. Quotes presented were selected for poignancy and representativeness of themes.

## Results

Nine semi-structured interviews were conducted between January and March 2014. Participant characteristics can be seen in Table 1. The average age of the mothers was 28.3 years (range 20–40 years), and the youngest infant or child in the household at the time of the interview was on average 12.2 months (range 6–22 months).

**Table 1:** Participant characteristics.

Participant	Smoking during pregnancy	Current smoking status at time of interview	Mother’s age at time of interview	Where home smoking took place	Employment	Mother’s report of partner’s smoking status	Child/family characteristics	Age of youngest child at time of interview
1	Quit for duration of pregnancy	Occasional smoker	26	Outside home	Employed	Smoker, separated	1 child; baby from PLS	6 months
2	Quit for duration of pregnancy	Daily smoker	30	Inside home, kitchen	Unemployed	No partner	4 children; baby from PLS and 3 older siblings	11 months
3	Quit for duration of pregnancy	Occasional smoker	23	Outside	Employed	Smoker	1 child; baby from PLS	11 months
4	Quit for duration of pregnancy	Regular smoker (smoked most days)	20	Outside	Unemployed	Smoker	1 child; baby from PLS	7 months
5	Relapsed during pregnancy	Daily smoker	24	Outside	Maternity leave	Smoker	5 children; 2 babies from PLS (twins), 1 older sibling, 2 younger siblings (twins)	7 months
6	Quit for duration of pregnancy	Occasional smoker	36	Outside	Employed	Smoker	1 child; baby from PLS	22 months
7	Relapsed during pregnancy	Daily smoker	30	Outside	Employed	Smoker	1 child; baby from PLS	17 months
8	Relapsed during pregnancy	Regular smoker (smoked most days)	40	Outside	Employed	Smoker	2 children; baby from PLS and 1 older sibling	15 months
9	Quit in final 2–3 months of pregnancy	Ex-smoker, currently only smoking electronic cigarettes	26	Previously smoked inside in kitchen. Currently smoked e-cigarettes throughout house	Employed	Ex-smoker, currently only smoking electronic cigarettes	2 children; baby from PLS and 1 older sibling	14 months

PLS—Pregnancy Lifestyle Survey

### Overview of findings

Mothers gave accounts of their smoking behaviour in pregnancy, and described how this changed as they progressed into the postpartum period. Mothers’ smoking intentions spanned across pregnancy and the postpartum period; however, these were adjusted over time reflecting their transient nature.

### Desire to be a responsible mother

Being a ‘responsible mother’ was interpreted as important in women’s accounts of smoking in pregnancy, with protecting the health of their baby cited as a primary motivation for quitting. Mothers had some awareness that smoking posed a risk to their baby during pregnancy, and used this knowledge to help inform their decision to quit:

*“I didn’t want to cause her [baby] any harm*. *I know the risk of smoking and I know obviously it can harm your baby*. *I’d wanted her for a long time*, *she was planned and I really wanted her so I wanted to make sure that obviously I gave her the best start.”*

(Participant 3)

Mothers who had not been able to stay quit during pregnancy described smoking in a way that managed potential risks to their baby within a level that they personally found acceptable, for example, by only smoking half a cigarette or not smoking every day. These risk reduction strategies were a compromise which enabled them to reconcile their current smoking, or their cravings, with their intentions to be a responsible mother by quitting.

*“I decided to quit smoking and then I kind of I did cut down*, *I didn’t smoke that much anyway in the first place*, *but I did really cut down*. *I think I stopped for a couple of months and then I’d have kind of the odd one.”*

(Participant 5)

The desire to be perceived by others as a responsible mother was also important; some mothers wanted to be responsible but did not necessarily want to quit; however, they felt pressured by others to do so:

*“I used to do it [smoke] behind his [partner] back sometimes*! *Which is wrong really because it’s only me that’s the bad one because I’m the one that’s carrying*, *you know*, *throughout the pregnancy sort of thing*, *so I’m only sort of*, *like*, *lying to myself really rather than lying to other people ‘oh no I’ve completely packed up’ but I hadn't*, *you know*, *I was having the odd one … I did feel bad but*, *you know*, *he’s [baby] turned out OK.”*

(Participant 8)

Similar to the prenatal period, being a ‘responsible mother’ was dominant in mothers’ accounts of their baby’s SHS exposure in the postpartum period. Some mothers approached this idea when describing how they had relapsed after giving birth:

*“When you become a mum you feel like you should be a lot more grown up … not just do it because everyone else is doing [it]*, *not just because I was drunk—but I thought ‘she’s [baby] not even with me*, *I'm having my first night away’ so I was enjoying myself and it’s not like I’d come home—she’d come home to me tomorrow and I will [not] still be stinking of them because obviously I would have got a shower and everything by then.”*

(Participant 3)

#### Anti-smoking attitudes towards smoking in pregnancy

Mothers held strong negative stereotypes about women who smoked during pregnancy:

*“I see so many people come in [to her place of work] and they’re heavily pregnant and would go out for cigarettes and I just think it is gross*. *[Laughter] I just think if you can’t quit for your own children what can you do for them*? *If you can’t quit for your unborn child—and plus if something was to happen to them you’d blame yourself wouldn’t you—you’d feel guilty—yeah*, *I don’t agree with it at all.”*

(Participant 3)

Women reinforced their position of being a ‘responsible mother’ by drawing on examples of other, less responsible, mothers who smoked whilst pregnant, even though they may have been smoking themselves since their baby had arrived. In doing so, mothers were able to compare and evaluate their own smoking behaviour favourably to others:

*“The girl [an acquaintance] I mentioned earlier who was smoking when she was pregnant and I found that quite disgusting and she still smokes now that she’s had the baby*, *so she’s not that bothered by smoking around her child*, *which I think is a bit disappointing*, *I mean*, *he [her acquaintance’s baby] doesn’t have a choice.”*

(Participant 6)

“My best friend … she smokes around her child—she smoked all the way through her pregnancy and she smokes around her son as well.”

(Participant 9)

These types of references to the ‘worse’ smoking behaviour of others, both in pregnancy and as smoking parents, were used to position themselves in a more positive light compared to others, and to help maintain their desire to be perceived as a responsible parent.

### Postpartum smoking; reasons or justifications for returning to smoking

Differences were observed in mothers’ described smoking trajectories postpartum. Of the five mothers that quit for the duration of pregnancy, two relapsed to smoking within a couple of days after giving birth and reported returning to pre-pregnancy smoking levels or higher. Three mothers who quit for the duration of pregnancy reported relapsing between two and six months postpartum, often whilst out with friends or socialising, and tended to describe occasional smoking thereafter. All mothers in this sample had prospectively reported smoking at 3 months postpartum; differences in retrospective reporting of the timing of their return to smoking may be due to memory/recall biases, or reflect differences in when mother’s first had a cigarette postpartum and when mothers considered themselves to have returned to smoking. Of the four mothers who had not quit for the duration of their pregnancy, two smoked within a couple of days after giving birth, and two mothers returned to smoking within two months of giving birth. These mothers reported returning to pre-pregnancy smoking levels. Despite these differing smoking trajectories, similarities were observed in how mothers repositioned their smoking status and identity as a smoker and a new mother.

Stress was a common theme in mother’s narratives of relapse, particularly for those who relapsed in the immediate postpartum period:

*“I had her on the Wednesday and she became quite poorly and I didn’t come out of hospital until the Sunday*, *and I was that upset in the hospital*, *I think I had one on the Saturday*. *I actually went outside the smoking centre entrance*, *which is disgusting*, *isn’t it*? *And I felt really*, *really bad*, *‘cause my ankles were as big as anything*, *I still had my jelly belly*, *and then*, *like*, *to me*, *people probably looked—I was still pregnant*. *Do you know what I mean*? *Which I didn’t like*. *But that was*, *again*, *because I couldn’t cope with the stress of her not being very well.”*

(Participant 7)

For those mothers who relapsed later in the postpartum period, being in a social situation with other smokers, or drinking alcohol, was commonly discussed as a trigger for relapsing:

“I didn’t want to start back up [smoking] but then I think he [her baby] was about 3 months old and I was able to go out and then just had the odd one [drink] and then started again.”

(Participant 8)

Some mothers attributed their relapse to habit, rather than addiction:

*“It [relapsing] was habit*, *habit*. *Because I’m not addicted to smoking*, *I was never addicted to it.”*

(Participant 1)

Postpartum, mothers described feeling *‘differently’* about smoking, with a subsequent change in risk perceptions. They were less concerned about the health implications for their baby as there was no risk of exposure in-utero:

*“After you’ve given birth then it’s*, *I kind of felt a bit differently about it [smoking] because then it wasn’t you know*, *affecting them [the babies].”*

(Participant 5)

#### Efforts or desire to reduce baby’s SHS exposure

Mothers described strategies employed to prevent or minimise SHS exposure for their baby in the postpartum period. Eight of the mothers described how their home was now smoke-free, with smoking taking place exclusively outside with the door shut:

*“Before obviously I was pregnant you just smoke in the front room sort of thing and then when other people used to visit it’s outside now*, *you know*, *from when I was pregnant because I said to my partner at the time ‘you’ve got to get used to going outside when [baby’s] born’ sort of thing so*, *you know*, *it’s a no smoking house now.”*

(Participant 8)

Pregnancy and parenthood were clearly an important life transition which precipitated attempts to make positive changes to mothers’ smoking; [18,19] whilst unsuccessful in remaining abstinent postpartum, the majority of mothers described their success in maintaining other positive changes in home smoking behaviours. Just one participant described currently smoking inside her home:

*“I just smoke in the kitchen with the back door open*. *That's it—I don’t smoke in any bedrooms or I don’t smoke in the living room—it’s just purely in the kitchen*. *Not while any of the kids are in there—just me on my own.”*

(Participant 2)

For this participant, the birth of her youngest child had caused her to change her home smoking behaviour; having previously smoked in the garden she now described smoking in the home. This participant’s description of her smoking in the home highlighted the barriers she experienced to smoking outside. For her, smoking in the kitchen was a compromise that allowed her to balance the safeguarding of her children whilst employing strategies (e.g. opening an external door) that she believed protected them from SHS exposure. This is linked to the theme ‘responsible mother’ as this participant describes doing the best she can to protect her children from SHS exposure given her circumstances as a single parent.

Other common strategies described by mothers to prevent exposing their baby to SHS included placing a time restriction between smoking and picking up their baby. This appeared to be pertinent for many of the interviewees, and for some enabled them to reduce the amount they smoked when they had childcare responsibilities:

*“I can remember*, *like*, *reading stuff saying that if you’ve had a cigarette you’re not allowed to go near them [baby] for half an hour and you’re not allowed to do this; you’re not allowed to do that*, *and I’m thinking*, *‘God*, *if I have a cigarette*, *I can’t even go and sit with her*.*’ So that stopped me a lot.”*

(Participant 7)

A final strategy described by mothers was acting as an advocate by protecting their baby from exposure to SHS from other people’s smoking. Advocacy was often described in one of two ways: either negotiations with others about their smoking behaviour, such as friends or family members, or through avoidance of situations in which they described a lack of agency to control others’ smoking, such as avoiding taking their baby to the homes of friends or family who smoked indoors:

*“I just said to everyone ‘you start washing your hands’ I've got a bottle of hand gel on the side of the back door and they have to use that*. *And I told them straight ‘you’ve got to smoke outside’ and I also told them that when we go to their house they need to smoke outside as well.’*

(Participant 3)

*“We’ve not spoken to this ‘friend’ [who smoked in her own home] since she’s [baby] been born … she [her friend] keeps asking us to go round there*, *and we’ve said maybe*, *‘cause we don’t want her [baby] to be in the smoke*, *whereas she don’t want to come round here ‘cause she don’t want to go outside for a cigarette*. *So that friendship’s died.”*

(Participant 7)

Mothers used these strategies as a way to manage any gap they experienced between their non-smoking intentions and their smoking behaviours postpartum; mothers had not been able to achieve their intention of being a non-smoker after having their baby and so employing these strategies enabled them to conform to their perception of a responsible mother through protecting their baby from SHS exposure, or reducing exposure to within a level that they found acceptable.

#### Anti-smoking attitudes towards parents who smoke

As with the strong anti-smoking attitudes towards women who smoke during pregnancy identified above, negative opinions towards smokers, in particular smoking parents, emerged during mothers’ later descriptions of their views about infant and child SHS exposure. These negative opinions were predominantly directed towards parents who smoked in the presence of their children; for many mothers a distinction was drawn between being a parent who smoked, and being a smoking parent who exposed their children to SHS, with the latter being considered irresponsible:

*“His [the baby’s father] sister and husband*, *they smoke around the children and she’s just had a baby and I think it’s disgusting… I really don’t like it*. *It makes me feel sick when I think of them smoking around their children and a newborn baby*, *smoking in the car non-stop*, *it just makes me feel so bad.”*

(Participant 1)

However, holding strong anti-smoking opinions towards smoking parents who exposed their children to SHS was for other mothers was in direct contradiction to their own smoking behaviour. Participant 2, who smoked in the kitchen of her home, described her shame at her own smoking, and her “disgust” at parents who smoked around their children:

*“It’s disgusting*. *I'm quite ashamed that I do smoke*. *I look at other people that walk along doing that or walk along with their toddlers and they start smoking and it looks absolutely disgusting and how they can breathe it all over their kids is just beyond me*. *And regardless of that even if I go out on my own if I’ve got a baby sitter I still wouldn’t smoke on the streets*, *I just don’t like it.”*

(Participant 2)

In her account above, she seems aware of the negative stereotype held towards parents who smoke, and describes avoiding smoking in public in an attempt to distance herself from this stereotype. Participant 7 similarly struggled to reconcile her anti-smoking attitudes with her own smoking, which resulted in her expressing her sense of disgust in herself:

*“I’d never go out in public with one*, *and when I was at work I never had one*, *because I were disgusted in myself and I don’t like seeing other people doing it.”*

(Participant 7)

### Repositioning smoking identity

Since relapsing to smoking either during pregnancy or following the birth of their baby, many mothers repositioned their smoking behaviour and adopted a new identity of an ‘social’ or ‘occasional’ smoker. This was driven by a reduction in the number of cigarettes women smoked; mothers drew comparisons between their smoking prior to pregnancy and postpartum to emphasize the change, illustrating that their own perception of their smoking had essentially changed since having their baby:

*“I was a full time proper smoker—like at work I’d go out for cigarette breaks and yeah—wake up in the morning—but now yes—and to go for none—but then I don’t ever fancy one*, *my boyfriend goes out for one and I don’t ever—I smell it on him but I don’t think ‘oh*, *I want one’.”*

(Participant 3)

*“I can go days with not having one and it’s only if I go out*, *you know*, *to socialise sort of thing that I decide to have one.”*

(Participant 8)

Smoking was considered to be on a continuum, whereby occasional smoking was both distinct from, and more acceptable than, being a regular *‘proper’* smoker. What was important for mothers was that they employed strategies to protect their baby from SHS exposure, and it was this which differentiated them from other smoking parents, or from the negative social stereotype of parents who smoke.

Repositioning of smoking behaviour and identity was also observed among mothers who otherwise reported themselves to be daily smokers. Participant 7 reports daily smoking of between 5–10 cigarettes per day, however similarly discusses how since having her baby she feels less dependent on smoking, is able to abstain from smoking for longer periods of time during the day, and tends to only smoke half of a cigarette rather than a full cigarette.

*“I can go all day without having one*. *So she’s changed me a lot for that … I don’t panic if I’ve not got any … if I can’t go out all day and get one*, *it doesn’t bother me.”*

(Participant 7)

*“If I go and have one*, *I’ll only smoke half of one*, *whereas before I used to smoke a full one.”*

(Participant 7)

While this mother did not describe herself as a ‘social’ or ‘occasional’ smoker, she was keen to illustrate differences between her smoking behaviour prior to pregnancy and since having her baby.

### Intentions

Mother’s smoking intentions appeared to be important in both the prenatal and postpartum period. All mothers described their intention to quit at least for the duration of their pregnancy, with those who relapsed before giving birth making further quit attempts as their pregnancy progressed:

*“I was about 5 or 6 months when I would have the odd one [cigarette] and then when I got towards the end I was like oh no*, *you know*, *better stop this*, *but I shouldn't have started it anyway*, *you know.”*

*(Participant 8)*.

The majority of mothers described their intentions to quit smoking not only for the duration of their pregnancy, but also permanently:

“I thought what’s the point of going 9 months—or 8 months not having one and then starting again afterwards—that's just pointless.”

(Participant 3)

This intention was influenced by several perceived factors, including knowledge or awareness of the risks associated with smoking and SHS; their desire to be a responsible mother; their desire to be perceived by others to be a responsible mother, and internalised negative attitudes towards women who smoked during pregnancy or parents who smoked around their children. However, all interviewees had relapsed to smoking by three months after the birth of their baby, with mothers’ intentions transitioning as a result of unsuccessfully staying quit. The strategies outlined above, such as placing restrictions on where and when they smoked and repositioning their smoking identity, reflected mothers’ new intentions to balance smoking with being a responsible mother. Whilst some mothers were satisfied with using these balancing strategies and had no further intentions to quit, others reiterated their intention to stop smoking permanently:

*“So that is my plan*, *is to stop [smoking] again*. *I can do it*, *I've got the willpower*, *just need to stop going out basically!”*

(Participant 8)

## Discussion

The results suggest that the desire to be, and/or to be perceived to be, as a ‘responsible mother’ were central to mothers’ accounts of their smoking behaviours during pregnancy and the early postpartum period. This was demonstrated in mothers’ descriptions of the harm reduction strategies they employed to protect their baby from SHS exposure, and their strong anti-smoking attitudes towards other smoking parents despite being smokers themselves. A key novel finding from this study was that after relapsing, mothers appeared to reposition themselves as ‘social’ or ‘occasional’ smokers rather than ‘regular’ smokers as they described themselves prior to pregnancy to align with their ideal of being a responsible mother.

### Strengths and Limitations

This is the first study, as far as the authors are aware, which explores the experiences and beliefs of mothers who abstained from smoking for at least part of their pregnancy but subsequently relapsed in the early postpartum period. A strength was the utilisation of one-to-one interviews, which facilitated in-depth discussion of home smoking experiences, behaviours and beliefs among a target group of mothers. Furthermore, these interviews were conducted in mother’s homes, which enabled the researcher to gain insight into the home environment and how this may contribute to their home smoking behaviours. Although only nine participants were sampled from a small cohort of mothers and interviewed, this was appropriate for obtaining a detailed interpretative account from this specific group. The personal characteristics of SO positioned the interviewer as an ‘outsider-researcher’, [26] which may have been influential throughout both the data collection and analysis process; the assumptions made and subsequent interpretation of interviewee’s accounts may be different to those made by either an ‘insider-researcher’, or someone who does not have background knowledge about smoking in pregnancy or child SHS exposure.

### Comparisons to previous literature

The reasons or circumstances mothers in this sample described around their return to smoking were similar to those reported in a recent qualitative synthesis [7] of experiences of postpartum relapse; stress, social influences and no longer needing to protect the baby from their smoking in utero were commonly cited when talking about their smoking relapse. The previous qualitative synthesis [7] of the evidence highlighted that research in this area was currently restricted to America and Canada, however, our findings illustrate that these issues are also pertinent within a UK sample.

The intention to be, or perceived by others to be, a ‘responsible mother’ dominated mothers’ narratives. Coxhead and Rhodes [27] similarly found smoking mothers of older children with respiratory illness were keen to portray themselves as ‘responsible smokers’ and ‘good mothers’, using emotive narratives and describing self-imposed smoking restrictions to demonstrate their good moral character. A strategy used by mothers in this sample was to draw on examples of other women or parents who smoked, demonstrating strong anti-smoking attitudes. Previous research has shown that individuals frequently reference either identifiable or generalised ‘others’ as part of forming moral tales and narrating experiences.[28] Comparisons to ‘others’ have been observed among smoking parents of older children (aged 0–19 years) to demonstrate who they identify themselves with, who they can make judgements of, and also to anticipate judgements of their own behaviour.[28,29] Mothers’ anti-smoking attitudes are likely to be influenced by ‘shared’ or ‘normative’ morals, [29,30] which predict both intentions and behaviour.[30] Moral tales of what is acceptable parental smoking behaviour are informed by community endorsements of smoking practices, and through comparisons to the worse smoking of ‘others’ help defend mothers’ own smoking behaviour.[29]

For some mothers in this sample, the desire to be a responsible mother, anti-smoking attitudes and normative morals towards smoking resulted in a sense of shame and disgust in their own smoking. As smoking rates continue to fall in the UK [31] those who do smoke are often in the minority, and report feeling a sense of stigma.[32] Many women who smoke during or soon after pregnancy will be from adverse sociodemographic backgrounds, [13,25,33,34] live with other smokers, [3,35] have greater interpersonal problems, [13] and may experience a lack of agency to change their smoking or the smoking of those around them. [36–41] A sense of shame and guilt among this group may further reduce self-efficacy to make positive changes to their smoking behaviour, or smoking in their homes. Future interventions need to be sensitive to the issues these mothers face, and avoid alienating this potentially vulnerable group by increasing their sense of shame and disgust about their smoking.

As found in previous research among older children [29,42] of smoking parents, mothers used harm reduction strategies to reduce or prevent SHS exposure for their baby. The most common of these was to make the home smoke-free, described by all but one in the current sample. This is in contrast to previous research, where mothers who relapse to smoking postpartum were vigilant in reducing their baby’s SHS exposure; [10,11] however, did not necessarily describe making their homes smoke-free.[11,43] Enforcing smoke-free rules often means negotiating with other smokers to implement restrictions, which can be challenging as it may be dependent upon equity in relationships with partners, family members or friends.[36–41] Similar to this previous research, [36–41] some mothers discussed a lack of agency, giving examples of sacrificing relationships where smoking restrictions could not be controlled in the homes of others. Some mothers in the current sample, however, were interpreted as having the agency to implement these restrictions in their own homes and the homes of others, such as family members, which may reflect greater community endorsement of protecting babies and infants from SHS exposure. A further issue described by mothers in the present study was the difficulty of balancing smoking with caring for a newborn baby, with some mothers describing their concerns of feeling they should leave a certain amount of time between smoking and being in close proximity with their baby. Future research is needed to explore this further, and any implications this may have for bonding with the baby and potential impacts on breastfeeding.

The repositioning of smoking identity in the postpartum period from being a ‘smoker’ to an ‘social’ or ‘occasional’ smoker interpreted in this sample has not, to the authors’ knowledge, previously been observed. A recent survey [44] of adults in California, USA, explored a new emerging category of smokers, labelled as ‘non-identifying smokers’, who report having smoked at least once in the previous 30 days but do not consider themselves to be a smoker. This group was estimated to comprise around 12.3% of all smokers in California. Non-identifying smokers were associated with having been a prior daily smoker, and having greater perceived control over their smoking behaviour. The authors argued that future tobacco control interventions should target this emerging smoking behaviour pattern, particularly within groups where smoking is stigmatised, and enforce the message that there is no safe level of smoking.[44] Robinson and Holdsworth [45] have previously discussed the limitations of the tendency to label adults as either ‘smokers’ or ‘non-smokers’. These one-dimensional categories are argued to not fully encompass the complexity of smoking and how smoking fits into people’s lives.[45] In this sample, transitioning from a ‘smoker’ to ‘social’ or ‘occasional’ smoker helped mothers to distance themselves from the perceived negative stereotype of being a smoking parent, and identify with the more positive label of ‘non-smoker’ which was better aligned with their desire to be a ‘responsible mother’. The distancing suggests that these women may be more receptive to messages around cessation or behaviour changes, such as implementing smoking restrictions in their homes, and maintaining these over the longer term. However, this also has implications for future interventions, which need to be designed to take mother’s self-perceptions of their smoking identity into consideration.

### Implications

These findings can inform the development of future interventions to prevent or reduce infant and child SHS exposure in the home. These should incorporate mothers’ smoking self-identity; as mothers who relapse principally view themselves as ‘social’ or ‘occasional’ smokers, interventions that are highlighted as relevant for women with these types of smoking patterns are more likely to be responded to, and, ultimately, be effective. This may involve widening the criteria used to identify smokers to be more inclusive of occasional or social smoking behaviour patterns, and raising awareness that there is no safe level of smoking and even occasional smoking is harmful.[44,46] Anti-smoking attitudes and normative morals towards parents who smoke were influential in mothers’ accounts of their smoking behaviour, and their perception of being a responsible mother. Interventions that focus on strengthening a community’s normative morals to protect infants and young children from SHS exposure, for example, by increasing awareness about the dangers of exposing infants and children to SHS, are therefore also likely to be helpful. There is evidence that this can be achieved through person-to-person spread of changing smoking behaviour, which cascade to others within larger social networks.[47] However, such approaches should be sensitive to avoid exacerbating a sense of shame in smoking behaviour, as observed in the current sample. Finally, interventions should suggest actions that parents and families can take to reduce or prevent their child’s SHS exposure. These may build on existing strategies that families have already implemented, for example, increasing the length of time between smoking and interacting with their child, or help parents acquire new skills or strategies, for example negotiation skills to ensure family and friends conform to home smoking rules.

## Conclusions

Being a ‘responsible mother’ dominated mother’s accounts of their smoking behaviour; mothers described using strategies to protect their infant from SHS exposure, and held strong negative attitudes towards other smoking parents. After relapsing, mothers appeared to reposition themselves as ‘social’ or ‘occasional’ smokers rather than ‘regular’ smokers. These findings suggest that interventions to prevent/reduce infants' home SHS exposure should build on mothers' intentions to be responsible parents, and should be highlighted as relevant for mothers who view themselves as ‘social’ or ‘occasional’ smokers.

## Supporting Information

S1 Table Consolidated criteria for reporting qualitative studies (COREQ): 32-item checklist. (PDF)
