# Supplementary material for: “I Was a Full Time Proper Smoker”: A Qualitative Exploration of Smoking in the Home after Childbirth among Women Who Relapse Postpartum
Source: PLoS One. 2016 Jun 16;11(6):e0157525. doi: 10.1371/journal.pone.0157525 (PMC4911111; doi:10.1371/journal.pone.0157525)
Supplement: S1 Table — (PDF) [file pone.0157525.s001.pdf]

Consolidated criteria for reporting qualitative studies (COREQ): 32-item checklist

Developed from:

Tong A, Sainsbury P, Craig J. Consolidated criteria for reporting qualitative research (COREQ): a 32-item checklist for interviews and focus groups. *International Journal for Quality in Health Care*. 2007. Volume 19, Number 6: pp. 349 – 357

**No. Item**	**Guide questions/description**	
**Domain 1: Research team and reflexivity**		
Personal Characteristics		
1. Interviewer/facilitator	Which author/s conducted the inter view or focus group?	It is reported that SO conducted all interviews
2. Credentials	What were the researcher's credentials? E.g. PhD, MD	BSc MSc
3. Occupation	What was their occupation at the time of the study?	PhD Student, Research Fellow
4. Gender	Was the researcher male or female?	Female
5. Experience and training	What experience or training did the researcher have?	It is reported that SO had a background in health psychology and smoking in pregnancy. SO had an MSc and had previously conducted interviews with a range of different participant groups, including health care professionals, carers, healthcare students and individuals with learning disabilities. She also had experience of conducting focus groups.
Relationship with participants		
6. Relationship established	Was a relationship established prior to study commencement?	No
7. Participant knowledge of the interviewer	What did the participants know about the researcher? e.g. personal goals, reasons for doing the research	It is reported that all participants were informed with an information letter about the purpose of the study. Additionally, the study was explained at the time of interview.
8. Interviewer characteristics	What characteristics were reported about the interviewer/facilitator? e.g. Bias, assumptions, reasons and	It is reported that all interviews were conducted by SO, who is female and has a background in

	interests in the research topic	health psychology and smoking in pregnancy
**Domain 2: study design**		
Theoretical framework		
9. Methodological orientation and Theory	What methodological orientation was stated to underpin the study? e.g. grounded theory, discourse analysis, ethnography, phenomenology, content analysis	It is reported that Interpretative phenomenological analysis (IPA) was the approach used.
Participant selection		
10. Sampling	How were participants selected? e.g. purposive, convenience, consecutive, snowball	All sampling procedures are reported. Convenience sampling was used.
11. Method of approach	How were participants approached? e.g. face-to-face, telephone, mail, email	It is reported that participants were contacted by mail
12. Sample size	How many participants were in the study?	It is reported that the sample consists of nine participants.
13. Non-participation	How many people refused to participate or dropped out? Reasons?	It is reported that 37 of those invited did not participate. Reasons for this include decline to participate, ineligibility and non-response to interview invite.
Setting		
14. Setting of data collection	Where was the data collected? e.g. home, clinic, workplace	It is reported that interviews took place in participant's homes
15. Presence of non-participants	Was anyone else present besides the participants and researchers?	Participants were offered the opportunity to have a family member or friend present. No participants took this offer.
16. Description of sample	What are the important characteristics of the sample? e.g. demographic data, date	Age, education, marital status, partner smoking status, employment and occupation of main household income earner if applicable
Data collection		
17. Interview guide	Were questions, prompts, guides provided by the authors? Was it pilot tested?	It is reported that a semi-structured interview schedule guided the interview, with prompts used as necessary. This was pilot tested with a patient participant involvement member.
18. Repeat interviews	Were repeat inter views carried out?	No

	If yes, how many?	
19. Audio/visual recording	Did the research use audio or visual recording to collect the data?	It is reported that interviews were audio recorded.
20. Field notes	Were field notes made during and/or after the inter view or focus group?	No field notes were made during the interview. Field notes were made immediately after each interview.
21. Duration	What was the duration of the inter views or focus group?	It is reported that interviews lasted on average 40 minutes
22. Data saturation	Was data saturation discussed?	Data saturation is discussed.
23. Transcripts returned	Were transcripts returned to participants for comment and/or correction?	No
**Domain 3: analysis and findings**		
Data analysis		
24. Number of data coders	How many data coders coded the data?	It is reported that two authors, SO and LLJ, coded the data
25. Description of the coding tree	Did authors provide a description of the coding tree?	A coding tree was not explicitly used
26. Derivation of themes	Were themes identified in advance or derived from the data?	It is reported that analysis was inductive; themes were derived from the study.
27. Software	What software, if applicable, was used to manage the data?	It is reported that data were transcribed verbatim into Word documents by professional transcribers. NVivo was used to facilitate coding.
28. Participant checking	Did participants provide feedback on the findings?	No
Reporting		
29. Quotations presented	Were participant quotations presented to illustrate the themes/findings? Was each quotation identified? e.g. participant number	Quotations are presented to illustrate the themes, identified by participant number
30. Data and findings consistent	Was there consistency between the data presented and the findings?	Yes
31. Clarity of major themes	Were major themes clearly presented in the findings?	Yes
32. Clarity of minor themes	Is there a description of diverse cases or discussion of minor themes?	Yes
